# Comparison of Dynamic and Liver-Specific Gadoxetic Acid Contrast-Enhanced MRI versus Apparent Diffusion Coefficients

**DOI:** 10.1371/journal.pone.0061898

**Published:** 2013-06-21

**Authors:** John N. Morelli, Henrik J. Michaely, Mathias M. Meyer, Thassilo Rustemeyer, Stefan O. Schoenberg, Ulrike I. Attenberger

**Affiliations:** 1 Department of Radiology, Scott and White Hospital, Texas A&M Health Sciences Center, Temple, Texas, United States of America; 2 Department of Clinical Radiology and Nuclear Medicine, University Medical Center Mannheim, Institute of Clinical Radiology and Nuclear Medicine, Mannheim, Baden-Württemberg, Germany; University Hospital of Essen, Germany

## Abstract

**Background:**

Hepatic lesions often present diagnostic connundrums with conventional MR techniques. Hepatobiliary phase contrast-enhanced imaging with gadoxetic acid can aid in the characterization of such lesions. However, quantitative measures describing late-phase enhancement must be assessed relative to their accuracy of hepatic lesion classification.

Purpose: To compare quantitative parameters in gadoxetic acid contrast-enhanced dynamic and hepatobiliary phase imaging versus apparent diffusion coefficients in hepatic lesion characterization.

**Material and Methods:**

57 patients with focal hepatic lesions on gadoxetic acid MR were included. Lesion enhancement at standard post-contrast time points and in the hepatobiliary phase (HB; 15 and 25 minutes post-contrast) was assessed via calculation of contrast (CR) and enhancement ratios (ER). Apparent diffusion coefficient (ADC) values were also obtained. Values for these parameters were compared among lesions and ROC analyses performed.

Results: HB enhancement was greatest with FNH and adenomas. HB ER parameters but not HB CR could distinguish HCC from benign entities (0.9 ER ROC AUC versus 0.5 CR ROC AUC). There was no statistically significant difference found between the 15 and 25 minutes HB time points in detection of any lesion (p>0.4). ADC values were statistically significantly higher with hemangiomas (p<0.05) without greater accuracy in lesion detection relative to HB phase parameters.

**Conclusion:**

Hepatobiliary phase gadoxetic acid contrast-enhanced MR characterizes focal hepatic lesions more accurately than ADC and conventional dynamic post-contrast time point enhancement parameters. ER values are generally superior to CR. No discernible benefit of 25 minute versus 15 minute delayed imaging is demonstrated.

## Introduction

Conventional dynamic contrast-enhanced MRI (cDCE-MRI) suffers limitations in characterization of focal hepatic lesions secondary to overlapping enhancement characteristics or variable appearances of lesions [1], [2]. This shortcoming of cDCE-MRI has been improved through the utilization of additional hepatic MR imaging techniques such as diffusion-weighted imaging (DWI) [3] and hepatocyte-specific contrast agents [4].

Diffusion-weighted imaging (DWI) also aids in identification and classification of hepatic lesions [3], [5]–[7]. DWI allows analysis of tissue proton Brownian motion, providing a quantitative measure of such motion by an apparent diffusion coefficient (ADC). Characterization of a focal hepatic lesion by ADC values has been shown useful so far in the setting of detection of metastatic disease [8] and in the characterization of hepatic hemangiomas and cysts [3]. Previous studies utilizing supraparamagnetic iron oxide contrast-enhanced MR demonstrated the benefit of the addition of diffusion weighted images in HCC detection and evaluation [9], [10].

Gadoxetic acid disodium (Eovist; Bayer Healthcare Pharmaceuticals; Gd-EOB-DTPA) is a gadolinium chelate MR contrast agent with 50% hepatobiliary excretion which allows improved characterization of hepatic lesions. Specifically, lesions containing functioning hepatocytes, such as focal nodular hyperplasia (FNH), demonstrate enhancement on delayed (i.e. hepatobiliary) phase imaging with gadoxetic acid, relative to lesions without functioning hepatocytes such as hepatocellular carcinomas (HCC) or metastases [11].

In the daily clinical routine some cases, especially the differentiation of solitary hepatic masses such as adenoma, HCC, and FNH, remain diagnostic conundrums. In such cases, quantitative assessments of DWI and contrast-enhancement parameters may aid radiologists in establishing a diagnosis. However, several questions remain. The first is which quantified measures of Gd-EOB-DTPA hepatobiliary phase enhancement allow for accurate differentiation of hepatic lesions. Specifically, it must be determined which of the two measurements reported in the literature for CA uptake, ER or CR is more reliable [12]. Second, Gd-EOB-DTPA is more costly than standard contrast agents, so the question of whether quantitative late phase imaging significantly outperforms early dynamic imaging is relevant. Third, DWI is an important modalit which does not require contrast administration, a fact particularly relevant given the nephrogenic systemic fibrosis discussion. Quantitative measurements for the differentiation of HCCs, adenomas, and FNHs should thus be analyzed.

Therefore, the aim of this present study is to evaluate the relative accuracy of Gd EOB-DTPA contrast-enhanced early dynamic and hepatobiliary phase imaging as well as quantified diffusion weighted imaging in the characterization of four major focal hepatic lesions (HCC, FNH, adenomas, hemangiomas).

## Materials and Methods

### Ethics Statement

The local institutional review board of the University Medical Center Mannheim approved and oversaw this study. All patient studies were performed at that institution. Informed consent was obtained. This clinical investigation was conducted according to the principles of the Declaration of Helsinki.

### Study population

A total of 178 consecutive patients referred for Gd-EOB-DTPA MR imaging of the liver as part of routine clinical practice at the University Medical Center Mannheim from January 2008–February 2011 were included in this institutional review board (IRB) approved retrospective study analysis. Informed written consent was obtained. Inclusion criteria were the presence of one or more focal hepatic lesions—specifically focal nodular hyperplasia, hepatic adenoma, hemangioma or hepatocellular carcinoma. Of the patients examined, 57 (26 men, 31 women, mean age 53.6±14.5 years) met the criteria for inclusion into the study. Of these, 21 hemangiomas, 18 hepatocellular carcinomas (11 of which were evaluated in cirrhotic livers), 10 hepatic adenomas, and 8 cases of focal nodular hyperplasia were assessed. Of these, 1 hemangioma and 2 hepatocellular carcinomas, were confirmed histopathologically at our hospital. The other lesions were confirmed by the clinical course of disease as established by imaging and clinical followup, the latter assessed by review of the medical record. In most cases, followup multi-phase MRI or CT examinations were available for followup assessment of lesions. In cases where lesions were also followed by ultrasound, these results were incorporated into the establishment of the final clinical diagnosis. The mean follow-up time period was 794 days; the mean number of follow-up exams was 7. Especially for benign lesions like hemangiomas, which are not routinely biopsied, diagnosis was based on taking all MR sequences into account for lesion assessment. If more than one lesion was imaged, then only the largest lesion was evaluated in the analysis. This approach was chosen as the focus of the study was on lesion characterization through enhancement characteristics and not on lesion detection. Exclusion criteria were incomplete examinations as well as lesions in which lesion size was too small to allow accurate ROI analysis. Among the 121 patients excluded were those with cholangiocarcinomas (n = 4), liver cysts (n = 25), metastases (n = 8), cirrhotic nodules (n = 30) as well as those with incomplete/inadequate scans or unconfirmed lesions as per the criteria stated above (n = 54).

### MR protocol

All patients underwent a MR-exam with a standardized protocol on a single 1.5T MR-system (MAGNETOM AVANTO 32×76, Siemens Healthcare Sector, Erlangen, Germany). Pre-contrast single-shot echo planar diffusion weighted imaging was obtained utilizing a product EPI-sequence with b values of 0, 50, 400, and 800 s/mm^2^ (TR/TE 8260.4/75 ms, acquisition time 3∶45 min∶sec, matrix 192×150, FoV 379×308, parallel imaging acceleration factor 2) during free breathing. ADC-values were calculated by the scanner using a monoexponential fitting based on all four measured b-values. Pre- and post-contrast T1-weighted images following Gd-EOB-DTPA administration were then obtained. The Gd-EOB-DTPA dose was standardized at 0.025 mmol/kg bw. A 6 channel body-array coil was utilized in combination with 6 elements of the spine matrix coil. 3D Volume-Interpolated Breathhold Examination (VIBE) sequences (TR/TE 5.5/1.93 ms, FA 30°, acquisition time 21.1 sec, matrix 384×188, FoV 370×265, slice thickness 3 mm, voxel size 1.0×1.4×3 mm^3^, parallel imaging factor 2) were used to acquire pre-contrast, early-phase dynamic extracellular and hepatobiliary phase imaging. For early-phase dynamic contrast-enhanced imaging with Gd-EOB-DTPA, VIBE sequences were obtained at 25 seconds (arterial phase), 60 seconds (portal venous phase), and 80 seconds (venous phase). Hepatobiliary phase imaging was obtained at 15 (HB1) and 25 minutes (HB2) following contrast injection.

### Image evaluation

Region of interest (ROI) analysis was performed on the acquired MR images by a single observer blinded to study methodology and aims. Circular ROIs were drawn on an offline workstation MacPro (Apple, Cupertino, CA) running OsiriX (OsiriX 3.7.1, The OsiriX Foundation, Geneva, Switzerland) on the pre- and post-contrast T1w images and ADC maps. The maximum size of the ROIs was fitted to the size of a given lesion. Care was taken that the ROI size did not exceed the edges of the lesion to avoid partial volume influence on the measured signal intensity. Areas of necrosis were likewise avoided in ROI placement. An additional ROI similar in size was drawn in a region of adjacent normal-appearing hepatic parenchyma. Care was taken to avoid inclusion of hepatic arterial or (portal-)venous structures. ROI data was collected along with the final diagnosis in Microsoft Excel.

### Contrast-enhancement parameters

Two parameters have been proposed for the assessment of enhancement with hepatocyte-specific MR contrast agents [12]. The enhancement ratio (ER) is calculated at a given post-contrast phase as [13]:
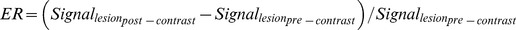
Enhancement ratios were calculated for each lesion, for each post-contrast time point evaluated. The contrast ratio (CR) has also been suggested as a measure of late-phase hepatobiliary enhancement and was calculated for each time point evaluated as follows [12]:
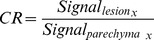
where x denotes a given pre- or post-contrast phase of imaging.

### Statistical analysis

Mean and standard errors about the mean were calculated for CR and ER measures at every time point post-contrast for each lesion type in SPSS (Statistical Processing for the Social Sciences v 13 SPSS Inc, IBM, Armok, NY). ADC values and signal values of normal hepatic parenchyma were similarly computed. One-way analysis of variance (ANOVA) measures were performed to test for differences among the lesions with respect to ER, CR, ADC values, and hepatic parenchymal signal. A Levene statistic to test for variance homogeneity was obtained. Multiple comparison tests were performed for each parameter to test for differences among the lesions depending on variance homogeneity for a given lesion. A Least Squares Differences test was utilized for multiple comparisons when variances were homogeneous and the Dunnett T3 in cases when variances were heterogeneous. P-values less than 0.05 were considered statistically significant for all comparisons.

ROC analyses assessing ER and CR were performed for each type of lesion at all available time points as well as for the ADC values. These analyses assessed whether a lesion was or was not of a certain type (i.e. a hemangioma or not). The analysis was performed in MedCalc for Windows version 11.6 (MedCalc Software, Mariakerke, Belgium). The area under each ROC curve (AUC) was obtained, and these values compared to the AUC of a diagonal curve (AUC = 0.5).

Comparisons of ROC measures generated by ER and CR values at both hepatobiliary phase time points were also performed utilizing MedCalc. Since the distinction between a diagnosis of HCC and not HCC is the most fundamental in this evaluation, the cutoff point which maximized combined sensitivity and specificity was identified on the ROC curve (the upper-most, left-most point) with MedCalc. For each lesion type, an additional comparison was analogously made between the early dynamic imaging phase with gadoxetic acid (pre-contrast, arterial, venous or portal venous) yielding the greatest absolute AUC value (utilizing either CR or ER) versus the hepatobiliary phase parameter with the greatest AUC value. Finally, hepatobiliary phase ER and CR AUC values were compared to those generated from ADC values for each lesion type.

The ability of hepatobiliary phase images (using both ER and CR values) and ADC values to differentiate between pairs of lesions was analogously compared. This was done utilizing pair-wise analyses of the 4 different lesion types (i.e. HCC, adenoma, hemangioma, FNH) resulting in a total of 6 comparisons. AUC values with ER, CR, and ADC values were compared to evaluate the ability of these measures to distinguish between pairs of lesions. The area under the ROC curves for each lesion were compared for hepatobiliary phase imaging and ADC values utilizing MedCalc as above. This was performed utilizing both CR and ER parameters. Early dynamic phase parameters were not utilized for the pairwise comparisons due to the decrease in statistical power attributable to making such a large number of comparisons and because the ROC analyses described in the previous paragraph had preliminarily shown equivalence or superiority of hepatobiliary phase versus early dynamic phase imaging.

## Results

### Mean comparisons

**Figure 1:**
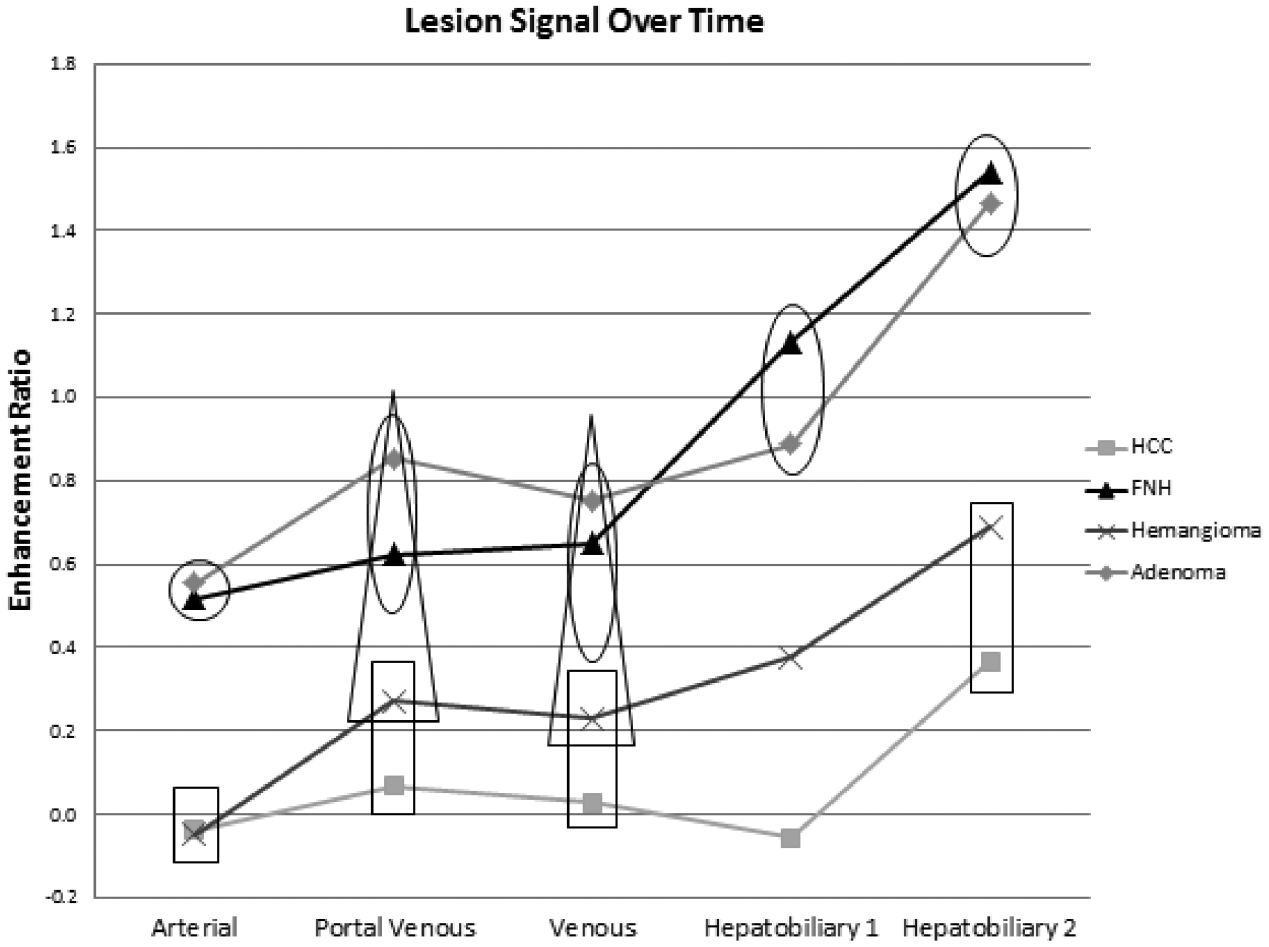
Mean enhancement ratios over time. Mean enhancement ratios not demonstrating statistically significant differences are enclosed within a single shape (i.e. circle, square, or triangle). Those not enclosed by the same shape demonstrate statistically significant differences in mean enhancement ratios for a given time point (p<0.05).

**Figure 2:**
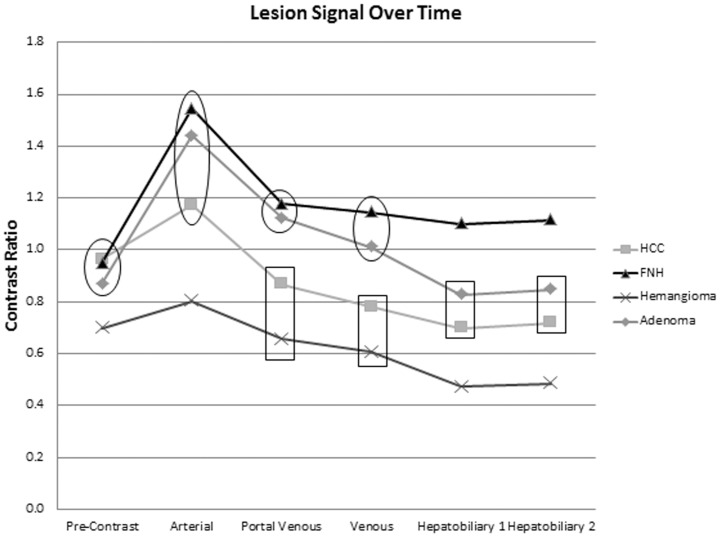
Mean contrast ratios over time. Mean contrast ratios not demonstrating statistically significant differences are enclosed within a single shape (i.e. circle, square, or triangle). Those not enclosed by the same shape demonstrate statistically significant differences in mean contrast ratios for a given time point (p<0.05).

The mean values for contrast (CR) and enhancement (ER) ratios of each lesion are depicted in Figures 1 and 2. The general trends were toward statistically significantly higher CR and ER with FNH and adenoma in HB1 and HB2 as compared to the other lesions.

Adenomas and FNHs could be differentiated with statistical significance from HCCs utilizing ER in HB1 and HB2. The mean (± standard deviation) ER of adenomas was 1.9±0.4 in HB1 and 2.5±0.7 in HB2 (p<0.05). The ER of FNHs was 2.1±0.4 in HB1 and 2.5±0.6 in HB 2 (p<0.05). HCC demonstrated lower HB1 and HB2 ER than all other lesions (mean HB1 ER = 0.9±0.3, mean HB2 ER = 1.4±0.7; p<0.001).

CR of FNH (1.1±0.2 at 15 minutes and 1.1±0.2 at 25 minutes) was statistically significantly higher than that of all other lesions (p<0.01; for 15 and 25 minutes, respectively: adenoma −0.8±0.2 and 0.8±0.2; hemangioma −0.5±0.1 and 0.5±0.2; HCC −0.7±0.2 and 0.7±0.2). Adenomas demonstrated significantly higher HB1 and HB2 CR than hemangiomas (p<0.01), but not HCC (p = 0.1).

**Figure 3:**
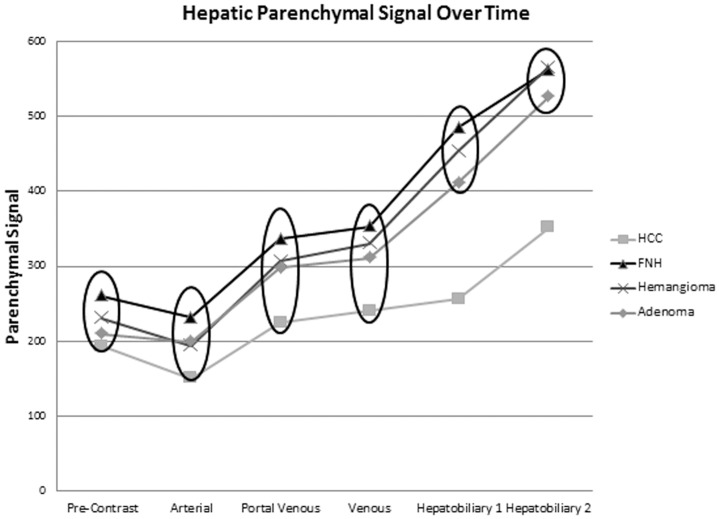
Mean hepatic parenchymal signal intensities over time. Mean parenchymal signal measures not demonstrating statistically significant differences are enclosed within a single shape (i.e. circle, square, or triangle). Those not enclosed by the same shape demonstrate statistically significant differences in mean parenchymal signal for a given time point (p<0.05).

**Figure 4:**
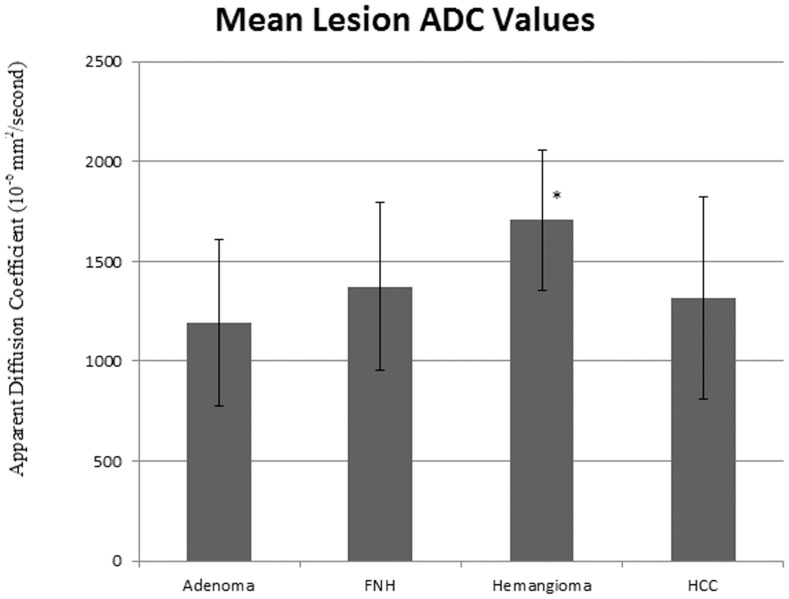
Mean lesion ADC values for each lesion type. * denotes p-values less than 0.05.

In the hepatobiliary phases, parenchyma of HCC livers demonstrated statistically significantly lower signal than all other livers (p<0.05) as illustrated in Figure 3. As shown in Figure 4, mean ADC values were higher with hemangiomas than with all other lesions (p<0.05).

### ROC analysis and determination of cut-off values

**Figure 5:**
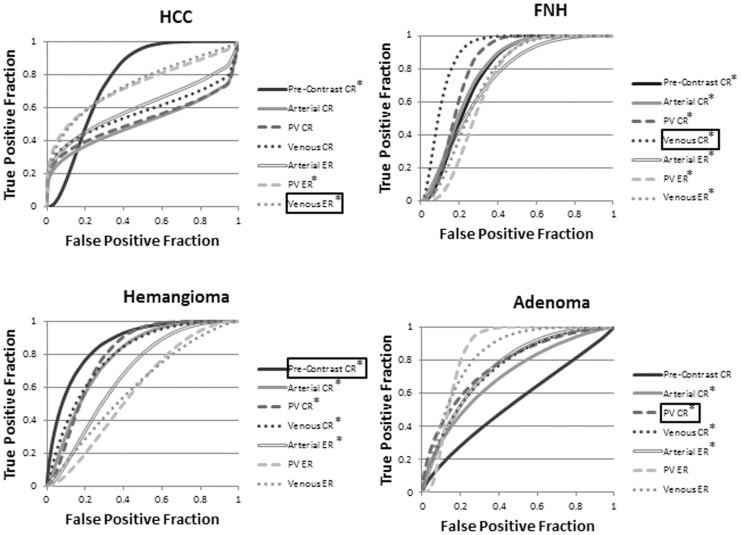
ROC curves generated from contrast ratio (CR) and enhancement ratio (ER) parameters. These were used for identification of hepatocellular carcinoma (HCC), focal nodular hyperplasia (FNH), hemangiomas, and adenomas in the pre-contrast, arterial, portal venous (PV), and venous phases. * reflects statistically significant (p<0.05) differences relative to a random test (AUC = 0.5). Parameters leading to the highest absolute AUC values are boxed; these parameters were utilized for comparisons with hepatobiliary phase imaging.

**Figure 6:**
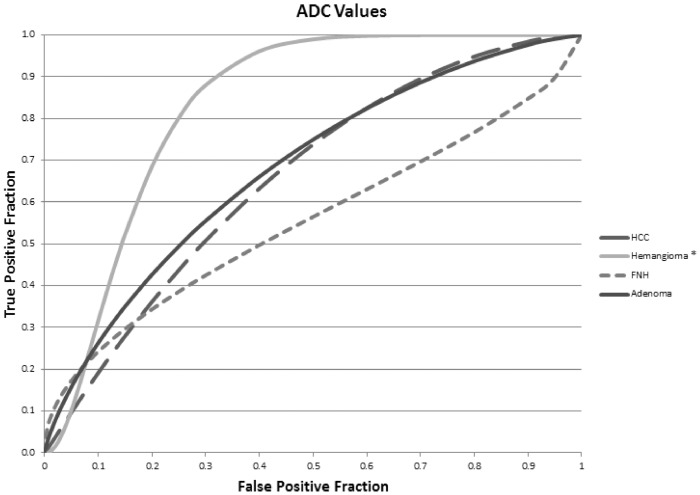
ROC curves for ADC values. These reflect the ability to detect hepatocellular carcinoma (HCC), focal nodular hyperplasia (FNH), hemangiomas, and adenomas. * reflects statistically significant (p = 0.05) differences relative to a random test (AUC = 0.5).

ROC curves for the differentiation of lesions by the early dynamic phase parameters (arterial – portal-venous – venous phase) are illustrated in Figure 5. The early dynamic phase-parameter combinations with the highest absolute AUC values were venous ER for HCCs (AUC = 0.8), venous CR for FNH (AUC = 0.9), pre-contrast CR values for hemangiomas (AUC = 0.9), and portal venous CR for adenomas (AUC = 0.8). ROC curves for lesion differentiation via ADC values are provided in Figure 6. Only hemangiomas were accurately identified by ADC values (AUC = 0.8, p<0.001).

**Figure 7:**
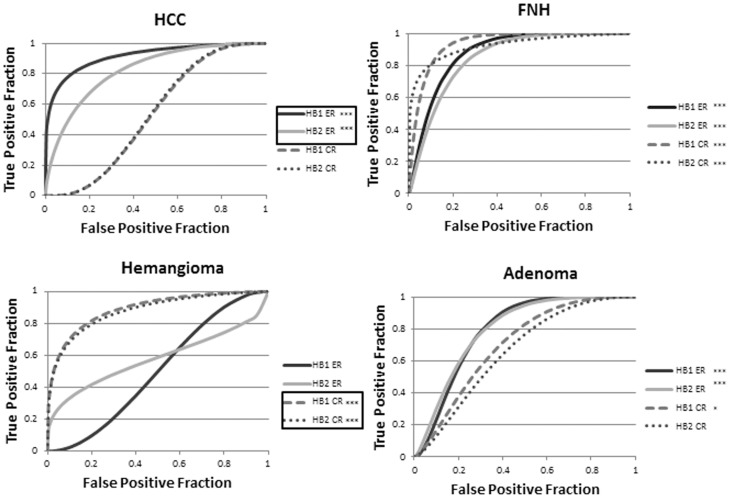
ROC curves generated from contrast ratio (CR) and enhancement ratio (ER) parameters. These were used for identification of hepatocellular carcinoma (HCC), focal nodular hyperplasia (FNH), hemangiomas, and adenomas at hepatobiliary phase time points 15 (HB1) and 25 minutes post-contrast. * reflects statistically significant (p<0.05) differences relative to a random test (AUC = 0.5) and *** reflects statistically significant reflects p-values less than 0.001. AUC values statistically significantly greater than others are boxed.

**Figure 8:**
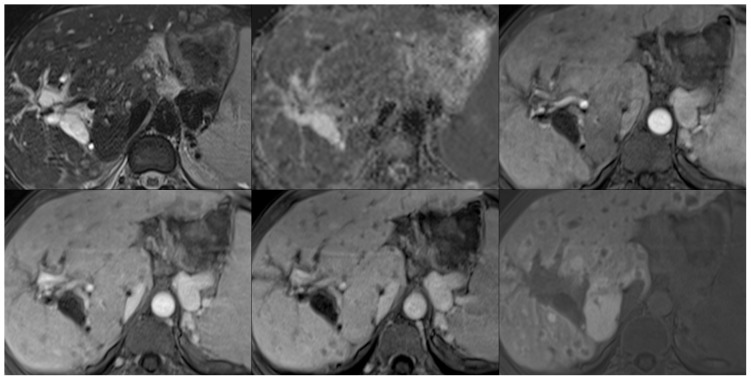
Representative images illustrating a case of hepatocellular carcinoma on fat saturated T2-weighted images (top left), ADC maps (top middle), and post-contrast images in the arterial (top right), portal venous (bottom left), venous (bottom middle), and hepatobiliary phases (15 minutes post-contrast; bottom right).

**Figure 9:**
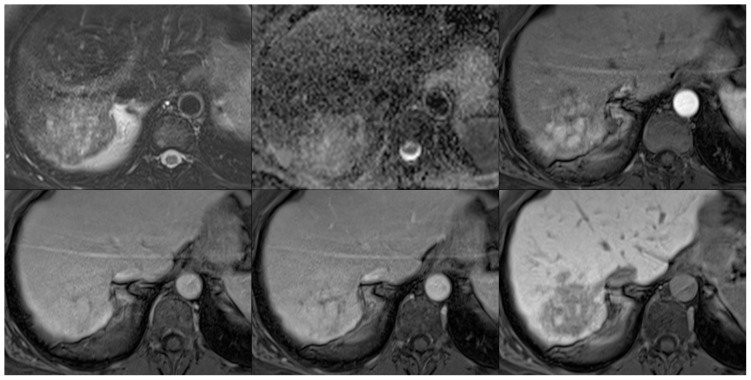
Representative images illustrating a case of focal nodular hyperplasia on fat saturated T2-weighted images (top left), ADC maps (top middle), and post-contrast images in the arterial (top right), portal venous (bottom left), venous (bottom middle), and hepatobiliary phases (15 minutes post-contrast; bottom right).

**Figure 10:**
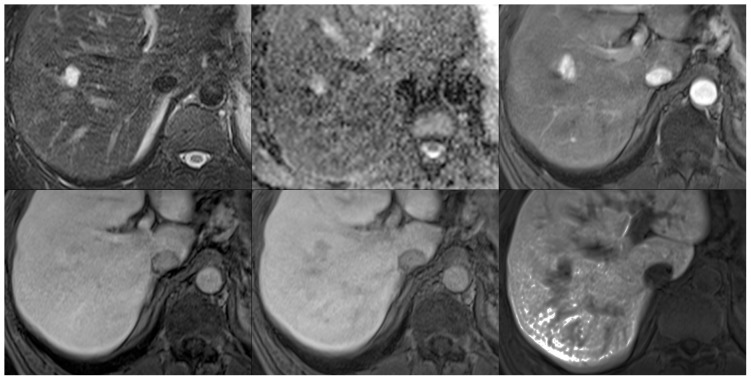
Representative images illustrating a case of a hemangioma on fat saturated T2-weighted images (top left), ADC maps (top middle), and post-contrast images in the arterial (top right), portal venous (bottom left), venous (bottom middle), and hepatobiliary phases (15 minutes post-contrast; bottom right).

**Figure 11:**
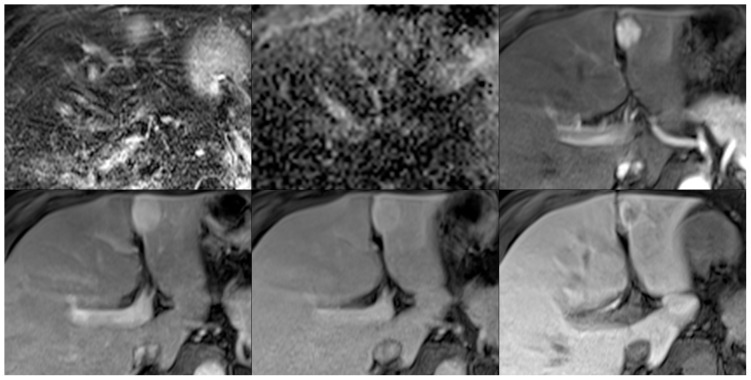
Representative images illustrating a case of hepatic adenoma on fat saturated T2-weighted images (top left), ADC maps (top middle), and post-contrast images in the arterial (top right), portal venous (bottom left), venous (bottom middle), and hepatobiliary phases (15 minutes post-contrast; bottom right).

The ROC AUC for hepatobiliary phase imaging is provided in Table 1 and depicted graphically in Figure 7. Comparing the assessed hepatobiliary phase enhancement parameters, ER was more accurate than CR in distinguishing HCC from non-HCC lesions (p<0.02), but CR was more accurate than ER at distinguishing hemangiomas from other lesions (p<0.001). No other statistically significant differences in the accuracy of CR and ER were found. In no case was the accuracy, as determined by AUC measures, of the 25 minute delayed hepatobiliary phase imaging statistically significantly different from that of the 15 minute delayed hepatobiliary imaging (p = 0.6). Examples of each type of pathology assessed in this study are provided in Figures 8–11.

**Table 1:** Area under the ROC curve for hepatobiliary phase (HB) imaging.

Lesion	Contrast Ratio		Enhancement Ratio	
	HB1	HB2	HB1	HB2
**HCC**	0.54	0.55	0.9♦	0.84♦
**FNH**	0.93♦	0.93♦	0.87♦	0.84♦
**Hemangioma**	0.89♦	0.88♦	0.51	0.55
**Adenoma**	0.70*	0.67	0.79♦	0.8♦

* denotes p-value<0.05 relative to a random test (AUC = 0.5).

♦ denotes p-value<0.001 relative to a random test (AUC = 0.5).

Bolded values denote AUC values significantly less than non-bolded values in a given row.

HB1, 2 = Hepatobiliary phase 1 and 2 (15 and 25 minutes post-contrast).

HCC = Hepatocellular carcinoma.

FNH = Focal Nodular Hyperplasia.

Comparing early dynamic versus hepatobiliary phase imaging, venous phase ER was less accurate than HB1 ER in the detection of HCC (p<0.01). In particular, specificity increased from 61.5% to 87% utilizing HB1 ER versus venous phase ER, while sensitivity remained constant at 94.4% utilizing a cut of values of 0.16. No other differences were found (p = 0.5).

Only in identification of hemangiomas, HB1 and HB2 ER were less accurate than were ADC values (p<0.005). No other significant differences were found between hepatobiliary phase parameters and ADC values (p = 0.3).

The AUC values for pair-wise lesion comparisons are provided in Table 2. Overall, ADC values allowed accurate differentiation between hemangiomas and all other lesions (p<0.05). However, in no case was the accuracy of ADC values statistically significantly greater than that of hepatobiliary phase enhancement parameters. Neither adenomas and HCC, nor FNH and HCC could be differentiated solely using ADC values, whereas hepatobiliary phase enhancement parameters allowed accurate differentiation. No statistically significant differences were seen between 15 and 25 minutes post-contrast hepatobiliary phase imaging for any pair-wise comparison. Hepatobiliary ER were statistically significantly more accurate than CR in the differentiation of adenomas versus HCC. No other statistically significant differences were found. Based on the ROC analysis, optimal cutoff values were determined for differentiation between HCC and benign lesions for hepatobiliary phase enhancement parameters and ADC values. These are provided in Table 3. Maximum sensitivity and specificity values obtained for quantitative conventional and hepatobiliary phase enhancement parameters for the detection of each lesion type are detailed in Table 4.

**Table 2:** Differentiation Between Pairs of Liver Lesions.

	Enhancement Ratio		Contrast Ratio		
ROC AUC	HB 1	HB 2	HB 1	HB 2	ADC
**FNH – Adenoma**	0.65	0.55	0.76*	0.8*	0.58
**FNH – Hemangioma**	0.88*	0.9*	1	0.99	0.83*
**FNH – HCC**	0.97*	0.94*	0.94*	0.92*	0.6
**Adenoma – Hemangioma**	0.8*	0.82*	0.9*	0.88*	0.87*
**Adenoma – HCC**	0.97*	0.93*	0.67	0.64	0.52
**Hemangioma – HCC**	0.86*	0.76*	0.83*	0.82*	0.8*

* denotes a statistically significant (p<0.05) areas under the curve values relative to a random test (AUC = 0.5).

Bolded values denote AUC values significantly less than non-bolded values in a given row.

HCC = Hepatocellular carcinoma.

FNH = Focal Nodular Hyperplasia.

ROC AUC = Receiver Operator Characteristic Area Under the Curve Values.

ADC = Apparent Diffusion Coefficient (mm^2^/s).

HB 1,2 = Hepatobiliary Phase (15 and 25 minutes post-contrast).

**Table 3:** Cutoff values for quantitative enhancement parameters in the differentiation of HCC from other solitary hepatic lesions.

	Optimal Cutoff	SS(%)	SP(%)
**HB1 ER** *	0.16	87.2	94.4
**HB2 ER** *	0.57	87.2	88.9
**HB1 CR**	0.49	88.9	38.5
**HB2 CR**	0.58	83.3	43.6
**ADC (mm^2^/s)**	1.19*10^−3^	61	87.2

* Denotes measures recommended quantitative parameter for this evaluation.

SS – Sensitivity.

SP– Specificity.

ER – enhancement ratio.

CR – contrast ratio.

HB 1,2 = Hepatobiliary Phase (15 and 25 minutes post-contrast).

**Table 4:** Maximum sensitivity (SS) and specificity (SP) values obtainable with quantitative conventional and hepatobiliary phase enhancement parameters.

Lesion	Parameter	SS (%)	SP (%)
**HCC**	ER venous	94	62
	ER HB	94	87
**FNH**	CR venous	100	67
	ER HB	100	78
**Hemangioma**	CR pre-contrast	91	89
	CR HB	76	89
**Adenoma**	CR portal-venous	70	72
	ER HB	90	67

ER – enhancement ratio.

CR – contrast ratio.

HB – hepatobiliary phase.

HCC = Hepatocellular carcinoma.

FNH = Focal Nodular Hyperplasia.

## Discussion

Accurate characterization of focal hepatic lesions is imperative due to resulting alterations in therapy and can obviate the risk of unnecessary biopsy or surgeries in the case of benign lesions. Likewise accurate identification of malignant lesions facilitates prompt treatment. MRI has emerged as a valuable tool in this regard, particularly with respect to hepatocyte-specific MR contrast agents and diffusion-weighted imaging. The present study examines focal hepatic lesion characterization with the latter two approaches. The results herein indicate that quantitative evaluations of hepatobiliary phase imaging with gadoxetic acid are useful in the characterization of several focal hepatic lesions, more so than ADC values or early dynamic phase imaging (i.e. pre-contrast, arterial, portal venous, and venous phase). In particular, HCC identification was improved with hepatobiliary phase ER measures compared with early-phase dynamic imaging.

Both CR and ER have been suggested as potential quantitative measures for hepatobiliary-phase enhancement with hepatocyte-specific contrast agents [12], and qualitative evaluations of hepatobiliary enhancement characteristics have relied on criteria analogous to CR [14]. No consensus currently exists as to whether CR or ER is a more reliable measure, a question addressed herein for the first time to the knowledge of the authors. This study finds that the principle drawback of the CR values are the inability to distinguish HCC from non-HCC lesions, relative to the ER parameter. This most likely relates to the diminished hepatobiliary phase parenchymal enhancement in patients with HCC, a finding previously shown in cirrhotic livers [15]. CR values for HCC are as a result elevated, making them similar to those values seen with adenomas. HCC ERs, on the other hand, remain decidedly lower than those of any other lesion. We thus conclude that CR values are less effective than ER values for identification of HCC; although, CR measures are more accurate in other specific situations such as distinguishing adenomas from FNH. However, the most critical decision in terms of the immediate therapeutic consequences for the patient, is to identify HCCs and to differentiate them from non-HCCs lesions. Thus, we would generally recommend the use of ER.

The utility of 25 minute versus 15 minute post-contrast hepatobiliary imaging is also a topic not previously studied in the literature to the knowledge of the authors. Previous authors have suggested 10–25 minutes post-contrast as the standard time point for hepatobiliary phase imaging with gadoxetic acid [16], [17]. In this study, no significant advantages of later phase hepatobiliary imaging (25 minutes post-contrast) were demonstrated in the identification of any particular lesion or differentiation between any two lesions. Reliance solely on 15 minute post-contrast hepatobiliary phase imaging with gadoxetic acid may improve practice throughput with the agent, a potential advantage of this compound over the other commonly utilized hepatocyte-specific contrast agent—gadobenate dimeglumine—with which hepatobiliary phase imaging is often performed with delays of up to 90–120 minutes [14], [18].

Previous studies have examined the benefit of DWI MR in the characterization of focal hepatic lesions: visual and quantitative assessments with DWI have been found useful in distinguishing hepatic hemangiomas and cysts from malignant hepatic lesions [3], [6], [19]; although, there is considerable overlap between the ADC values of solid benign (i.e. FNH and adenoma) and malignant lesions [5]. Differentiating hemangiomas from other focal hepatic lesions with gadoxetic acid enhanced MR has proven somewhat problematic in prior works: studies have suggested the lack of a true equilibrium phase with gadoxetic acid results in apparent early washout (i.e. pseudowashout) with hemangiomas [20], [21]. In fact, the early-phase parameter with the quantitatively greatest AUC value for hemangioma detection in our study was pre-contrast CR, which reflects that quantified measures of contrast enhancement are not helpful for the characterization of hemangiomas. Hemangiomas could rather be differentiated best on basis of quantitative measures using ADC values., which have been shown in this study as a way to improve upon these potential limitations of gadoxetic acid enhanced MRI. Although, ADC values were more accurate than ER values in distinguishing between hemangiomas and non-hemangiomas, there was no statistical difference in accuracy between ADC and CR values in this regard. In terms of pair-wise comparisons, ADC values did not significantly differ from hepatobiliary CR or ER values in accuracy. Results herein are consistent with prior works showing increased ADC values with hemangiomas relative to the other evaluated hepatic lesions; however, it is not clear if such values offer improved characterization of hemangiomas versus hepatobiliary phase imaging.

Limitations of this study include the inherent weaknesses associated with retrospective analyses. In particular, not all hepatic lesions were included in the analysis. Metastatic lesions were excluded because they have several diagnostic drawbacks: first, their small size results in partial volume effects deteriorating quality of quantified measures. Second, in retrospective analysis metastatic lesions would in many cases already be undergoing adjuvant chemotherapy treatment which could alter enhancement characteristics. Finally, metastases from different primary tumors exhibit different early-phase enhancement characteristics rendering results difficult to generalize.

A relatively small number of lesions assessed herein were confirmed histopathologically; unfortunately, this limitation is necessary to some extent as histological confirmation of benign hepatic lesions is typically not clinically necessary and thus unethical given the risks of biopsy to the patient. For differentiation of some lesions, this poses a particular problem. For example, even with long-term clinical follow-up and assessment of all available MRI sequences, it may be difficult or impossible to distinguish hepatic adenomas from FNH in certain cases. A recent study by Grazioli et al reported, with some heterogeneity, hypointensity of hepatic adenomas to parenchyma in the hepatobiliary phase [22]. Of note, the majority (6 of 10) of adenomas in our work were hypointense to hepatic parenchyma in the HB phase, and FNH and adenomas were accurately distinguished on the basis of CRs. The relatively greater degree of HB ER with adenomas observed herein (although, still less than mean FNH ER) may be due to the relatively low number of adenomas included in this study and/or reflect a greater proportion of “unclassified subtype” lesions, which are less likely to be hypointense in the HB phase [22]. It is possible that inaccurate clinical characterization of some adenomas as FNH's and vice versa could to contribute to this as well. Furthermore, the relative lack of cases with histopathological correlation poses a potential limitation with respect to evaluation of HCC with gadoxetic acid contrast-enhanced MRI. Specifically, a recent study by Kim et al revealed a greater degree of hepatobiliary phase enhancement in well-differentiated relative to moderately and poorly differentiated HCC [23]. As detailed assessment of HCC grade was not able to be performed in our work, the effect of HCC differentiation on our results is not known. It is possible that hepatobiliary phase imaging with gadoxetic acid would more accurately distinguish moderate and poorly differentiated HCC from the benign entities demonstrating increased hepatobiliary enhancement in this work (i.e. FNH and hepatic adenomas), whereas differentiation between these entities and well-differentiated HCC may prove more difficult. Finally, addition of qualitative parameters in the assessment of the liver lesions would likely aid in diagnostic accuracy. For example, a ring-enhancement pattern on gadoxetic acid contrast-enhanced MR has been shown to make the diagnosis of hemangioma less likely [21]. However, such qualitative assessments are inherently subjective. A radiologist may utilize a purely quantitative measure to gain objective data regarding a solitary hepatic lesion. In this case, knowledge of the relative diagnostic accuracy of the CR and ER values presented herein provide the practicing radiologist with quantitative data which can be synthesized with the qualitative interpretation to make a more confident diagnosis. In the case when a radiologist has narrowed the considerations between two focal lesions, pair-wise parameters may help determine between them.

In summary, quantified analysis of gadoxetic acid contrast-enhanced MR characterizes commonly encountered focal hepatic lesions more accurately than ADC values. Hepatobiliary phase imaging outperforms early dynamic imaging in the differentiation between HCC and non-HCC. Utilization of enhancement ratio values to characterize lesions is generally recommended over CR values.
